# Experimental Cross-Species Infection of Common Marmosets by Titi Monkey Adenovirus

**DOI:** 10.1371/journal.pone.0068558

**Published:** 2013-07-24

**Authors:** Guixia Yu, Shigeo Yagi, Ricardo Carrion, Eunice C. Chen, Maria Liu, Kathleen M. Brasky, Robert E. Lanford, Kristi R. Kelly, Karen L. Bales, David P. Schnurr, Don R. Canfield, Jean L. Patterson, Charles Y. Chiu

**Affiliations:** 1 Department of Laboratory Medicine, University of California San Francisco, San Francisco, California, United States of America; 2 UCSF-Abbott Viral Diagnostics and Discovery Center, San Francisco, California, United States of America; 3 Viral and Rickettsial Disease Laboratory, California Department of Public Health, Richmond, California, United States of America; 4 Texas Biomedical Research Institute, San Antonio, Texas, United States of America; 5 Southwest National Primate Research Center, San Antonio, Texas, United States of America; 6 California National Primate Research Center, Davis, California, United States of America; 7 Department of Psychology, University of California Davis, Davis, California, United States of America; 8 Department of Medicine, Division of Infectious Diseases, University of California San Francisco, San Francisco, California, United States of America; French National Centre for Scientific Research, France

## Abstract

Adenoviruses are DNA viruses that infect a number of vertebrate hosts and are associated with both sporadic and epidemic disease in humans. We previously identified a novel adenovirus, titi monkey adenovirus (TMAdV), as the cause of a fulminant pneumonia outbreak in a colony of titi monkeys (*Callicebus cupreus*) at a national primate center in 2009. Serological evidence of infection by TMAdV was also found in a human researcher at the facility and household family member, raising concerns for potential cross-species transmission of the virus. Here we present experimental evidence of cross-species TMAdV infection in common marmosets (*Callithrix jacchus*). Nasal inoculation of a cell cultured-adapted TMAdV strain into three marmosets produced an acute, mild respiratory illness characterized by low-grade fever, reduced activity, anorexia, and sneezing. An increase in virus-specific neutralization antibody titers accompanied the development of clinical signs. Although serially collected nasal swabs were positive for TMAdV for at least 8 days, all 3 infected marmosets spontaneously recovered by day 12 post-inoculation, and persistence of the virus in tissues could not be established. Thus, the pathogenesis of experimental inoculation of TMAdV in common marmosets resembled the mild, self-limiting respiratory infection typically seen in immunocompetent human hosts rather than the rapidly progressive, fatal pneumonia observed in 19 of 23 titi monkeys during the prior 2009 outbreak. These findings further establish the potential for adenovirus cross-species transmission and provide the basis for development of a monkey model useful for assessing the zoonotic potential of adenoviruses.

## Introduction

Adenoviruses (AdVs) are non-enveloped icosahedral double-stranded DNA viruses that infect a number of vertebrate hosts, including humans and nonhuman primates. The genus *Mastadenovirus* within the *Adenoviridae* family includes 7 human adenoviral species A-G (HAdV-A through HAdV-G) and 1 simian adenoviral species A (SAdV-A) [1]. In humans, infections by adenoviruses cause conjunctivitis, gastroenteritis, hepatitis, myocarditis, and acute respiratory illness, ranging from the “common cold” syndrome to fatal outbreaks of pneumonia [2], [3]. However, existing animal models of adenovirus infection to date have been primarily confined to rodents [4], [5], and no nonhuman primate (NHP) model has been established to study adenoviruses that infect humans and/or NHPs.

We previously identified a novel adenovirus, titi monkey adenovirus (TMAdV) in association with a fatal outbreak of pneumonia and hepatitis in a closed colony of captive New World titi monkeys (*Callicebus cupreus*), with evidence for potential cross-species transmission to a human researcher and family member with concurrent acute respiratory symptoms [6]. The origin and natural host reservoir for TMAdV remained unknown, although neutralizing antibodies to TMAdV were found in 23 titi monkeys demonstrating clinical signs, 14 titi monkeys exposed to animals demonstrating clinical signs, 1 rhesus macaque (*Macaca mulatta*), and the 2 humans. Studies by other groups have also revealed the widespread presence of closely related adenoviruses in both human and nonhuman primates [7], [8], [9], and large-scale serological surveys have detected antibodies to monkey adenoviruses in humans living in endemic regions [10], [11]. In addition, human adenovirus species E and G each contain only one member isolated from humans [12], [13], with the remaining members all isolated from monkeys or apes. Collectively, these data have raised concerns regarding the potential of adenoviruses as sources for emerging zoonotic disease in humans.

To further investigate the pathogenicity of the novel adenovirus TMAdV, we sought to develop an *in vivo* animal model of infection and disease for TMAdV. While TMAdV was originally discovered in a closed colony of captive titi monkeys (*Callicebus cupreus)* at the California National Primate Research Center (CNPRC), we recognized that *in vivo* testing in this monkey species was contraindicated due to the significant devastation to the colony caused by the virus. Since TMAdV was able to be successfully propagated in the marmoset lymphocyte cell line B95a *in vitro* (Table 1), we instead elected to pursue *in vivo* testing of TMAdV infection in the common marmoset (*Callithrix jacchus*).

**Table 1:** Tropism of TMAdV in human and monkey cells and cell lines.

Source	Name of Cell Line	Cell Type	Growth
Human	HEK293	human embryonic kidney	+
	CaCo-2	human epithelial colorectal adenocarcinoma	++
	A549	human epithelial lung adenocarcinoma	+++
	HFDL	human fetal diploid lung	+++
Monkey	PMK	primary rhesus monkey kidney (Old World monkey)	+++
	B95a	marmoset monkey lymphoblastoid (New World monkey)	++
	Vero	African green monkey kidney (Old World monkey)	+++
	BSC-1	African green monkey kidney (Old World monkey)	+++

+, weak cytopathic effect (CPE); ++, moderate CPE, +++, strong CPE.

The common marmoset is a New World primate that is small, easily handled, and highly susceptible to infectious agents, and thus a suitable nonhuman primate model for infectious disease [14], [15]. Marmosets have been successfully used to characterize a number of emerging viral diseases, including filovirus- and arenavirus-induced hemorrhagic fevers, encephalitis, and severe acute respiratory syndrome (SARS) [16]. Since titi monkeys (Family Pithecidae, subfamily Callicebiniae) and marmoset monkeys (Family Cebidae, subfamily Callitrichinae) are classified into separate families [17], the use of the marmoset for *in vivo* testing of TMAdV infection also afforded the opportunity to directly test the capacity of TMAdV, and, by extension, adenoviruses in general, to cross the species barrier and cause productive infection in a related yet taxonomically distinct secondary host.

## Results

### Cell Culture Adaptation of TMAdV

**Figure 1:**
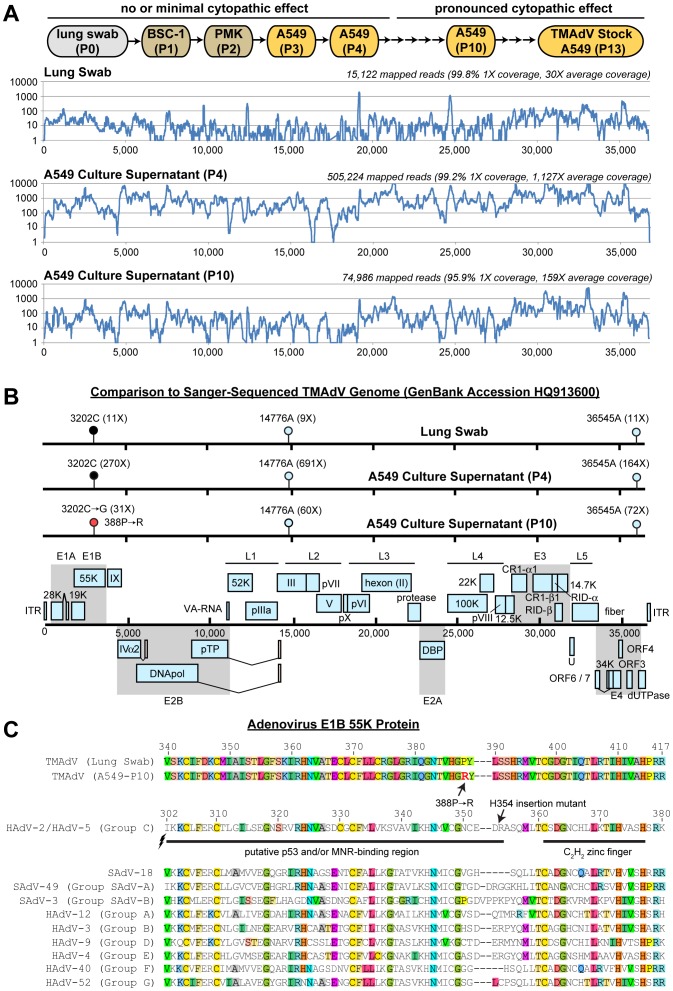
Whole-Genome Sequencing of Serially Passaged TMAdV. (A) Genomic coverage of TMAdV by mapping of deep sequencing reads from a lung swab from a titi monkey with TMAdV pneumonia, passage 4 in A549 cells, and passage 10 in A549 cells. The coverage (y-axis) achieved at each position along the ∼37 kB TMAdV genome (x-axis) is plotted on a logarithmic scale. (B) Identification of single nucleotide polymorphisms (SNPs) in assembled TMAdV genomes. The coverage at each SNP position is shown in parentheses. SNP positions 14776A and 36545A (cyan lollipops) correspond to probable sequencing errors in the Sanger sequenced TMAdV genome (Genbank HQ913600). A 3202CG mutation is observed to occur between passage 4 (black lollipop) and passage 10 (red lollipop), which results in a 388PR amino acid coding change in the TMAdV E1B-55K protein. (C) A multiple sequence alignment of the E1B-55K protein of TMAdV and representative human and simian adenoviruses reveals that position 388 resides within a putative binding region for p53 and/or the MRE11-RAD50-NBS1 (MRN) complex. Human adenovirus type 5 (HAdV-5) mutants containing a nearby H534 insertion or mutations in the downstream C_2_H_2_ zinc finger inhibit binding to p53 and/or MRN [18].

Previously, we were only able to successfully propagate TMAdV in the A549 human lung adenocarcinoma cell line [6]. Viral adaptation, as assessed by robust cytopathic effect (CPE), occurred by passage 6, and, while the wild-type or early passaged strains grew poorly in available human and monkey cells, the fully adapted passage 10 was able to efficiently infect both types of cells (Fig. 1A) [6]. To understand the molecular basis behind this adaptation, unbiased next-generation, or “deep” sequencing was used to resequence the TMAdV genome corresponding to the original isolate, derived from a lung swab corresponding to a titi monkey who died of TMAdV pneumonia, and passages 4 and 10 in A549 cells (Fig. 1A). From approximately 16, 30, and 54 million 100-nt (“nucleotide”) reads generated per sample, 15,122, 74,986, and 505,224 reads corresponding to the original isolate, passage 4, and passage 10 (P10), respectively, were mapped to the TMAdV genome. A consensus genome was calculated from the mapped reads, and then scanned to identify SNPs, or single nucleotide polymorphisms, that differed from the Sanger-sequenced TMAdV genome (GenBank accession number HQ913600). In total, only 3 SNPs were detected between the Sanger-sequenced TMAdV genome and the 3 genomes assembled by deep sequencing (Fig. 1B). Two of the SNPs, 14776A and 36545A, were identical in all three TMAdV genomes assembled, and thus likely correspond to PCR and/or sequencing errors in the original Sanger-sequenced viral genome. The sole remaining SNP is a C→G mutation at position 3202 that results in a putative amino acid change from proline to arginine at position 388 of the TMAdV E1B-55k protein (388P→R). PCR, cloning, and sequencing of a short gene segment encompassing position 3202 at different viral passages confirmed the correlation between the presence of the 3202 C→G mutation at passages 7 through 13 and TMAdV adaptation in A549 cells beginning at passage 6 (Fig. 1A; Table S1). Since the adenoviral E1B-55K protein is known to interact with and degrade cellular DNA repair proteins such as p53 and the MRE11-RAD50-NBS1 (MRN) complex [18], [19], a multiple amino acid sequence alignment of E1B-55K proteins was performed between TMAdV and representative human and simian adenoviruses to determine whether the identified mutation might be involved (Fig. 1C). The 388P→R amino acid change was found to be located in a putative p53/MRN-binding region of the E1B-55K protein [18], [19].

Next, we attempted to propagate P10 TMAdV in a variety of human and monkey cell lines (Table 1). Productive infection as assessed by the magnitude of CPE was observed in all cell lines tested. Notably, moderate growth of TMAdV was seen using marmoset lymphoblastoid B95a cells, the only New World monkey cell line tested. Both human epithelial colorectal cells (CaCo-2) and lung cells (A549, HFDL) also supported TMAdV replication.

### Experimental Infection of Marmosets with TMAdV

**Figure 2:**
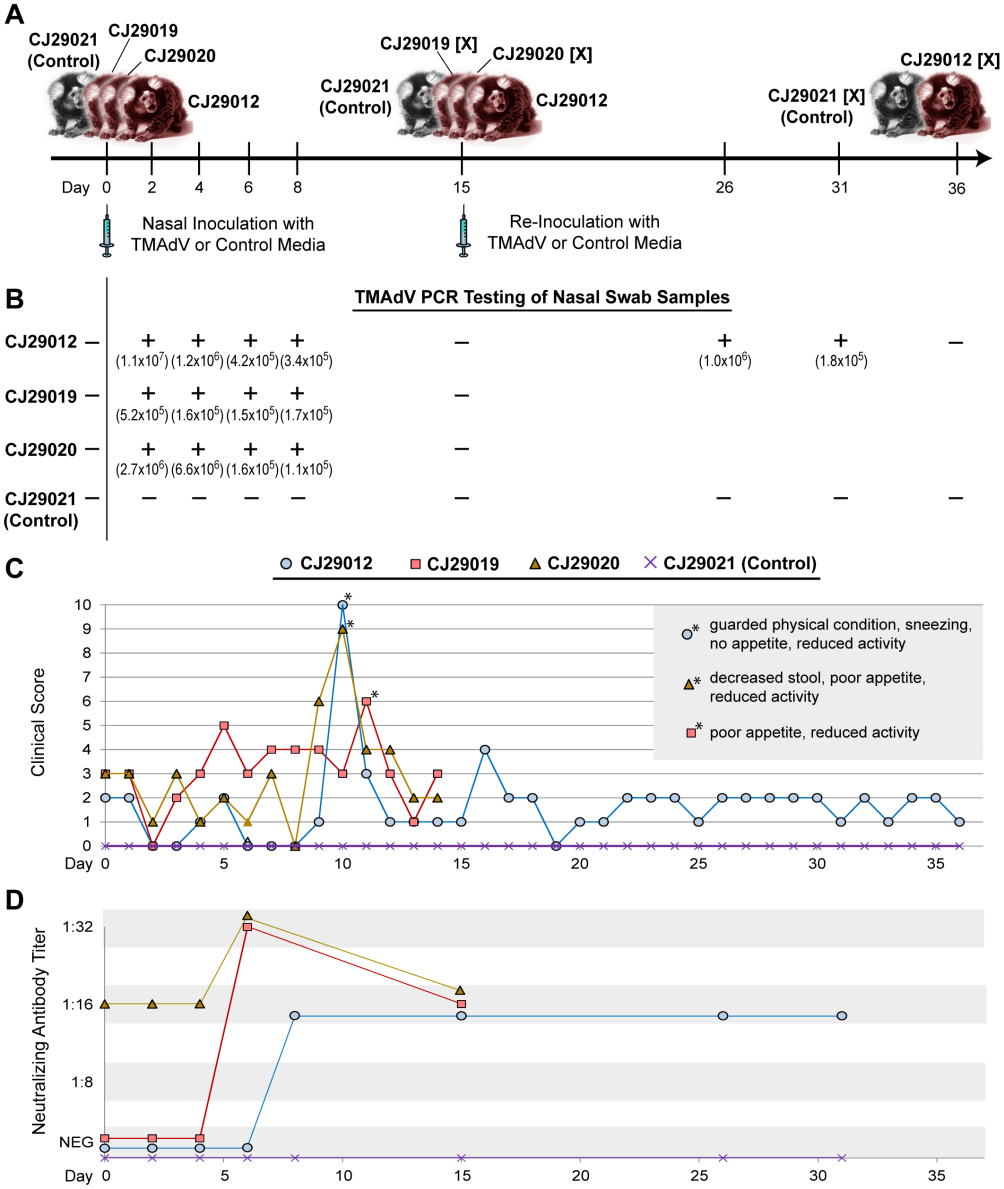
Experimental TMAdV Infection in the Common Marmoset. (A) Outline of TMAdV infection protocol and sample collections. The “[X]” refers to a marmoset that is sacrificed at the designated timepoint. (B) Results from PCR analysis of nasal swab samples collected at serial timepoints. Shown in parentheses are the calculated viral titers in genome copy numbers per mL associated with each timepoint. (C) Clinical scores in TMAdV-infected and control marmosets. The asterisks (*) refer to timepoints during which each infected marmoset exhibited the most pronounced clinical signs of illness (inset box). For a definition of the clinical scoring system, see Table S2. (D) Antibody titers in TMAdV-infected and control marmosets as measured using a TMAdV neutralization assay.

We examined TMAdV infectivity by inoculating marmosets intranasally with 0.1 mL of 10^5^ TCID_50_/mL of P10 virus for which sufficient viral stocks were available. The inoculation dose (10^4^ TCID_50_) was chosen to be similar to the physiologic doses used in experimental nasal inoculation of nonhuman primates at the TBRI with acute respiratory viruses such as RSV (respiratory syncytial virus). Three marmosets, CJ29012, CJ29019, and CJ29020, were inoculated with culture-adapted TMAdV (passage 13), while one marmoset, CJ29021, was inoculated with cell culture media as a control (Fig. 2A). All animals appeared normal until approximately 5 to 10 days after infection, when clinical signs presented in 3/3 inoculated monkeys (clinical scores >4) (Fig. 2C; Table S2). Animals exhibited reduced activity, had decreased stool production, became anorexic, and developed low-grade fevers. Marmoset CJ29012 demonstrated the most severe clinical signs and was in guarded physical condition with nasal congestion and sneezing by day 10. After day 11, all 3 experimentally infected animals recovered quickly, with marmosets CJ29019 and CJ29020 fully recovered at the time of euthanasia on day 15. To assess the effect of re-infection with TMAdV, marmoset CJ29012 was re-inoculated on day 15 and, along with control marmoset CJ29021, was observed for an additional 21 days prior to euthanasia at day 36. No clinical signs of infection were observed in marmoset CJ29012 (experimental TMAdV animal) or marmoset CJ29021 (experimental control animal) after re-inoculation (clinical scores < = 4).

### TMAdV Detection by PCR and Neutralizing Antibody Titers

Nasal swabs collected from the 3 experimental marmosets were positive for TMAdV by real-time qRT-PCR on 2, 4, 6, and 8 days post-inoculation, and negative by day 15 at the time of euthanasia (Fig. 2B). After re-inoculation, nasal swabs from marmoset CJ29012 were positive for TMAdV on days 26 and 31 after re-inoculation, but negative by day 36 at the time of euthanasia. Calculated viral titers ranged from 1.1×10^5^ to 1.1×10^7^ genome copies per mL by qRT-PCR (Fig. 2B). There was no evidence of viremia by qRT-PCR or nested PCR in serum samples from any of the marmosets studied (data not shown).

A virus neutralization assay was then used to screen experimentally infected marmosets for the development of neutralizing antibodies (Abs) to TMAdV (Fig. 2D). Interestingly, whereas marmosets CJ29012, CJ29019, and CJ29021 (control) each had negative pre-inoculation Ab titers of <1∶8, marmoset CJ29020 had a pre-existing antibody titer of 1∶16. Antibody titers in TMAdV-infected marmosets CJ29012 and CJ29019 rose to 1∶32 and 1∶16 by days 6 and 8 post-inoculation, respectively, whereas the antibody titer in marmoset CJ29020 rose slightly from 1∶16 to 1∶32 on day 6. Antibody titers remained constant at 1∶16 upon re-inoculation of marmoset CJ29012 on day 15 and during the entire 21-day follow-up observation period.

### Gross Pathology and Histology

**Figure 3:**
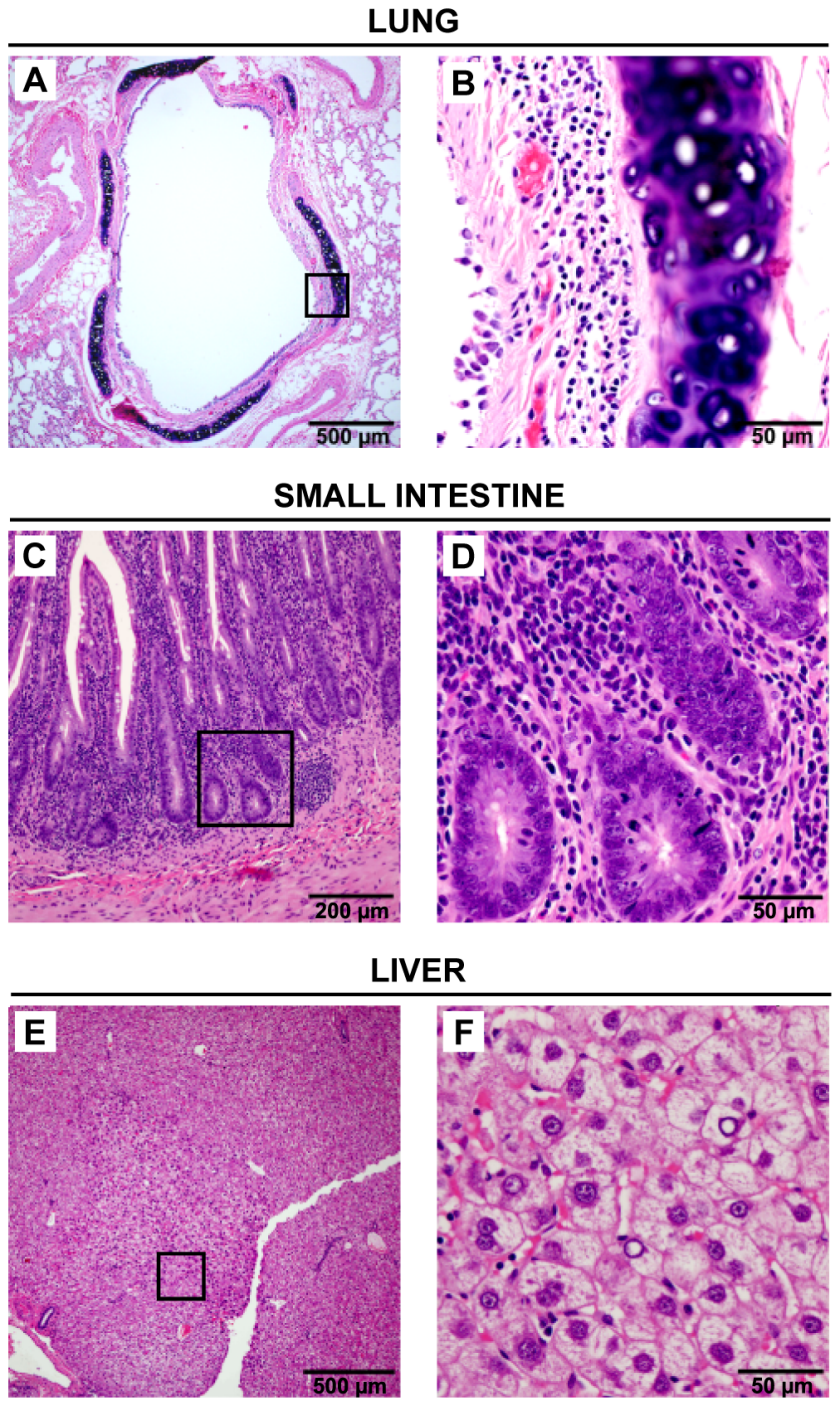
TMAdV-Associated Pathology in Experimentally Infected Marmoset CJ29019. Histologic findings on necropsy tissue from day 15 post-inoculation include a mild bronchitis (A and B), enteritis (C and D), and an atypical nodular hyperplasia in the liver (E and F). The black rectangle in panels A, C, or E outlines the region magnified in panels B, D, or F, respectively.

No significant gross pathological lesions were observed. Only a few salient histologic findings were specific to TMAdV-inoculated animals (Table 2). A mild bronchitis (Fig. 3A–B) and focal area of atypical nodular hyperplasia of the liver (Fig. 3E–F) were observed in marmoset CJ29019 (Table 2). All 3 inoculated animals were found to have a mild enteritis (Fig. 3C–D) and/or colitis. Typical basophilic intranuclear inclusions consistent with active adenovirus infection were not observed in tissues from any of the TMAdV-infected marmosets. The paucity of histologic findings observed in marmosets experimentally infected with TMAdV is in sharp contrast with the striking lesions observed in moribund titi monkeys with TMAdV-associated pneumonia and hepatitis (Table 2) [6].

**Table 2:** Salient histologic findings in TMAdV-infected marmoset and titi monkeys with rapidly fatal pneumonia.

Monkey	Tissue	Pathology	Intranuclear InclusionBodies	Finding for indicated animal	
				29012	29019
Marmoset	Ilver	Atypical nodular hyperplasia	absent	−	++
	lung	bronchitis	absent	−	+
	intestine	enteritis/colitis	absent	+	+
				titi monkey with pneumonia	
titi*	liver	hepatitis, necrosis	present	+++	
	lung	pneumonia, hemorrhage	present	+++	
	trachea	inflammation, destructionof normal architecture	present	+++	

+, mild; ++, moderate; +++, severe; −, absent.

* some titi monkeys developed only subclinical infection or mild acute respiratory illness from TMAdV, for which tissue is unavailable [6].

### Screening for TMAdV in Necropsy Tissues by Direct Immunofluorescence

**Figure 4:**
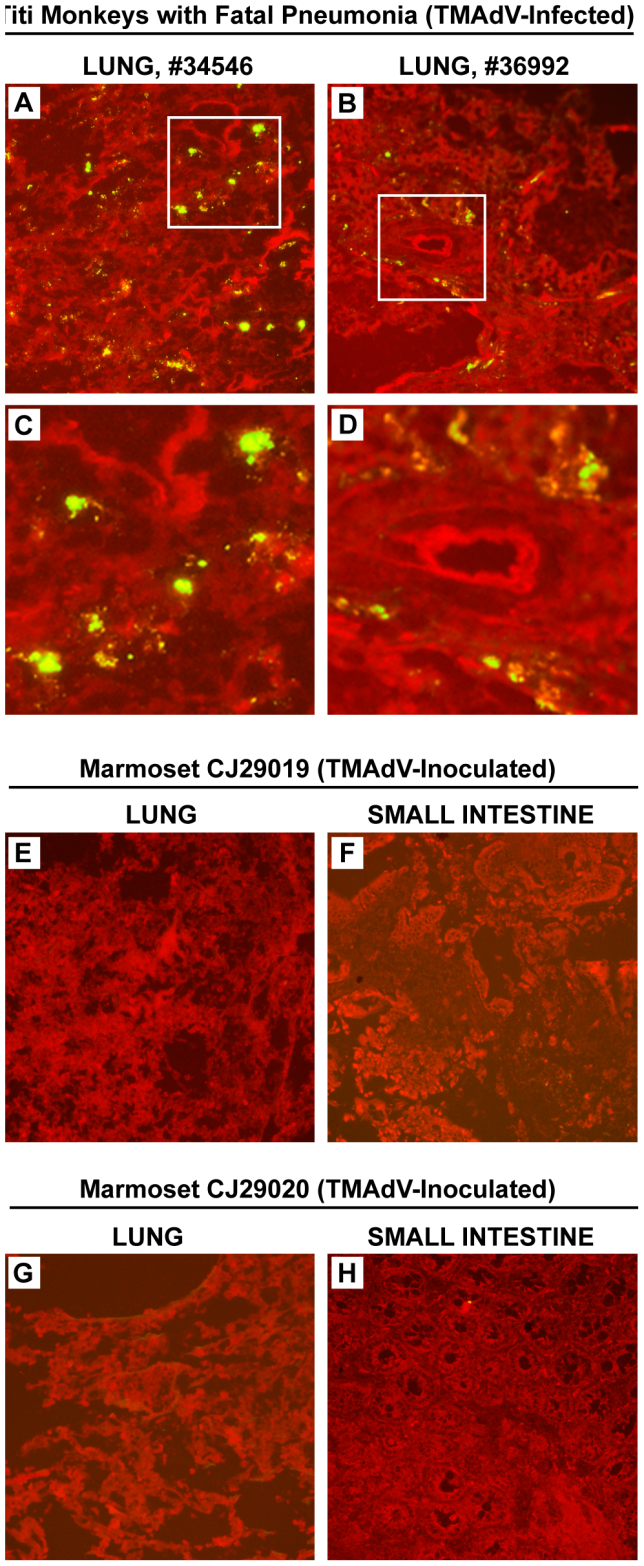
TMAdV Antibody Staining of Titi and Marmoset Monkey Tissues. Direct immunofluorescence staining of lung from two TMAdV-infected titi monkeys with fatal pneumonia (A-B, with corresponding magnified views in C-D) [6], and lung and liver from two TMAdV-inoculated marmosets (E-H) are shown. TMAdV staining is evident in lung from naturally infected titi monkeys who died from pneumonia, but absent in lung and liver from experimentally infected marmosets.

A direct immunofluorescence assay for broad detection of adenoviruses was used to screen for TMAdV in necropsy tissues from experimentally infected marmosets in the current study and naturally infected titi monkeys from the previously reported pneumonia outbreak [6]. Areas of bright apple-green fluorescence, predominantly cytoplasmic, were observed in lung tissues from titi monkeys with fatal TMAdV pneumonia (Fig. 4A–D), but not in lung or intestinal tissues from experimentally TMAdV-infected marmosets (Fig. 4E–H). To confirm these negative findings, necropsy marmoset tissues were then tested for the presence of TMAdV by real-time qPCR and nested PCR. TMAdV was not detected in necropsy tissues from any of the marmosets by these two PCR assays.

## Discussion

Here we present evidence supporting infection of common marmosets with TMAdV, a novel adenovirus previously associated with acute respiratory illness in humans and rapidly fatal hepatitis and pneumonia in titi monkeys [6]. Although we were unable to re-isolate the virus from blood or tissues from TMAdV-infected marmosets, nasal cultures were positive for TMAdV by real-time qRT-PCR for up to 15 days post-inoculation, and TMAdV inoculation produced both clinically apparent disease and antibody responses in inoculated animals. Thus, River’s modifications to Koch’s postulates, which recognize the additional serological evidence of antibody production in response to a viral infection, have been fulfilled [20], [21]. Experimental infection by nasal inoculation at a physiologic dose of 10^4^ TCID_50_ resulted in an acute, self-resolving “flu-like” illness starting around days 5 to 10 post-inoculation and was characterized by low-grade fever, reduced activity, decreased stool production, and anorexia; repeated episodes of sneezing were observed in 1 animal. This was accompanied by an increase in virus-specific neutralizing antibody titers in serum in all 3 inoculated animals, confirming an acute, productive yet self-limited infection by TMAdV in marmosets. The sole animal with pre-existing neutralizing antibody to TMAdV was a surprising finding given that we did not anticipate that infection by TMAdV or a serologically cross-reactive New World monkey adenovirus was prevalent among marmoset populations. Further investigation into this possibility is ongoing. The general lack of significant histologic lesions consistent with active adenovirus infection in inoculated animals (Table 2) may be a function of the delay between appearance of clinical signs and necropsy. Taken together, these results demonstrate that TMAdV is infectious and causes disease in marmosets, and clearly documents the ability of adenoviruses to cross species barriers in closely related hosts.

*In vivo* infection of marmosets with a physiologic dose of TMAdV was unable to replicate the rapidly fatal pneumonia and hepatitis syndrome seen in some of the titi monkeys infected with the virus during the pneumonia outbreak at the CNPRC (Table 3) [6], and necropsy tissues including lung, intestine, liver, were negative for TMAdV, albeit after complete resolution of clinical signs. Although the mild bronchitis seen in one TMAdV-infected marmoset (CJ29019) is consistent with acute respiratory infection, the enteritis and/or colitis seen in all 3 inoculated marmosets is most likely a manifestation of mild marmoset wasting syndrome, which is extremely common even in healthy marmoset populations [22], especially given the absence of intranuclear inclusion bodies and negative immunofluorescence results (Fig. 4). The difference in clinical outcome from TMAdV infection may also be related to anatomical differences between titi monkeys and common marmosets. In titi monkeys, enlargement of the larynx and the presence of laryngeal air sacs have been described [23], which may have predisposed the animals to aspiration pneumonia secondary to laryngeal swelling from viral infection. It is possible that evidence of more severe and invasive infection would have been observed by increasing the infectious dose or numbers of experimentally infected animals, or by serial sacrifice experiments. However, naturally acquired infections by adenoviruses are typically mild and fatal outcomes are rare in immunocompetent individuals [24]. Notably, clinical signs in more than one-third of titi monkeys documented to be infected by TMAdV during the previously reported pneumonia outbreak by antibody testing were also mild or absent [6].

**Table 3:** Differences in TMAdV-associated disease between marmosets, titi monkeys, and humans.

	Marmoset (n = 3)*	Titi Monkey (n = 37)**	Human (n = 2)***
mode of infection	experimental, with passaged TMAdV harboring a 3202C→G mutation	natural, with wild-type TMAdV	natural, with wild type TMAdV (probable zoonotic transmission)
clinical signs or symptoms	low grade-fever, reduced activity,decreased stool, anorexia, sneezing (n = 1)	fever, cough, lethargy, respiratorydistress (23 cases)	fever, cough, pleurisy
disease	brief respiratory illness with spontaneous recovery	severe pneumonia and hepatitis	prolonged (4 weeks) upper respiratory illness
subclinical/mild infection possible?	yes (100%, or 3 of 3)	yes (14 cases)	unknown
case fatality rate	0% (0 of 3)	83% (19 of 23 cases with pneumonia/hepatitis)	0% (0 of 2)
viremia present	no (0 of 3)	yes, rarely (2 of 6 fatal cases; 0 of 4survivors among cases examined)	unknown
necropsy findings	mild bronchitis and atypical nodular hyperplasia of the liver (1 of 3),mild enteritis and/or colitis (3 of 3)	diffuse, consolidated pneumonia with hemorrhage; hepatic necrosis and hemorrhage; destruction of normal lung, tracheal, and liver architecture (in fatal cases)	n/a
intranuclear inclusionbodies observed?	no (0 of 3)	yes (in fatal cases)	n/a
Immunofluorescent staining of lungand liver positive for TMAdV?	no (0 of 3)	yes (in fatal cases)	n/a
electron microscopy of lung positivefor adenoviral particles?	not done	yes (lung alveoli)	n/a
magnitude of antibody responseto TMAdV infection	1∶16–1∶32	up to >1∶512	1∶16 (researcher) and 1∶8 (household member)

* 3 marmosets experimentally infected with TMAdV in the current study.

** 23 cases of largely fatal pneumonia/hepatitis and 14 cases of subclinical TMAdV infection in titi monkeys at the California National Primate Research Center (CNPRC) [6].

*** TMAdV-infected human researcher and household member at the CNPRC [6].

Similar to the 3 inoculated marmosets in the present study, infection by TMAdV in a human researcher at the CNPRC and household member was associated with self-limited “flu-like” respiratory illness [6]. The observed peak levels of neutralizing antibodies (1∶16 to 1∶32) in response to TMAdV infection in marmosets from the current study, as well as in rhesus monkeys, titi monkeys, and humans with mild/absent clinical signs or symptoms during the CNPRC outbreak, were significantly decreased relative to those in titi monkeys with fulminant pneumonia (up to >1∶512) [6]. Levels of neutralizing antibody titers may thus correlate with disease severity, as seen for other respiratory diseases such as SARS [25], and this association may be related to the phenomenon of immune hyperactivity and “cytokine storm” in critically ill individuals.

Necropsy tissues tested negative for TMAdV by both PCR and immunofluorescence, and TMAdV was also absent in blood throughout the course of infection. The absence of significant viremia in TMAdV-infected marmosets is not surprising given that TMAdV viremia was rarely found even in titi monkeys with fatal pneumonia and hepatitis (Table 3). These results indicate that the respiratory tract, and not blood, may be the primary site of infection and replication by TMAdV. Indeed, in our current study, we were able to detect TMAdV in marmosets up to 2 weeks after post-inoculation in nasal swabs but not in blood.

Another potential explanation for the differences in TMAdV infection between marmosets or humans (acute, self-respiratory illness) and some titi monkeys (fulminant pneumonia) may have been attenuation of the virulence of the serially passaged inoculated strain by the lone 388P→R amino acid change in the adenoviral E1B-55K protein (Fig. 1; Table S1). Previously, we noted that adaptation of TMAdV to cell culture required 6 passages for robust growth in Old World monkey (rhesus and African green monkey) and human A549 cells [6]. The present study has now uncovered the molecular basis for that adaptation. We hypothesize that this single 388P→R coding alteration during passaging may have promoted growth and adaptation of TMAdV in culture, yet may have attenuated the infectivity and/or virulence of the virus *in vivo*. The clinical significance, if any, of this alteration is unclear; the original proline residue at that position can be found in simian, bovine, porcine, and tree shrew, but not human adenoviruses (data not shown), but the 388P→R change or an arginine at that position has not been noted in any sequenced adenovirus to date. Experiments to investigate this role of this amino acid change in infectivity are now underway.

The different manifestations of TMAdV-associated disease between naturally-infected titi monkeys and experimentally-infected marmosets are also not surprising given that they are members of two different families of nonhuman primates. For many viral infections, such as monkey herpesvirus B infection in humans and macaques [26], the spectrum and severity of the disease vary significantly between different, albeit related, hosts. In this study, productive infection of marmosets by TMAdV is documented by the clinical signs of illness beginning at days 5-10, specific neutralizing antibody responses, and detection of the virus in nasal swabs up to 2 weeks post-inoculation. These results along with additional data from recent literature [7], [8], [9], [27] now firmly establish that infection by adenoviruses can indeed cross taxonomic barriers within different nonhuman primate and human hosts. Additional research is needed to develop the common marmoset as a nonhuman primate model for adenovirus disease, especially from viral strains such as TMAdV with the potential to cause both fulminant illness and cross-species infection [6].

## Methods

### Animal Ethics Statement

This study was carried out in strict accordance with the recommendations in the Guide for the Care and Use of Laboratory Animals of the National Institutes of Health and under animal biosafety level 2 (BSL-2) conditions with personnel using BSL-3 practices as approved by the Institutional Biohazards Committee of the Texas Biomedical Research Institute (TBRI). The protocol for marmoset study 1305-CJ, “The pathogenicity of the novel adenovirus TMAdV in a marmoset animal model”, was approved by the Institutional Animal Care and Use Committee and the Institutional Biohazards Committee of the TBRI. TBRI is accredited by the Association for Assessment and Accreditation of Laboratory Animal Care (AAALAC) International and operates in accordance with the NIH and U.S. Department of Agriculture guidelines and the Animal Welfare Act. Marmosets were kept healthy and well-nourished with strict feeding protocols and close monitoring of their health status prior to the start of the study and during the entire study period. One week before inoculation, animals were transferred to the biosafety level-3/4 facility at the TBRI and housed individually in cages specifically developed for marmoset work. Marmosets were sedated and humanely euthanized by administration of a sodium pentobarbital solution by a licensed veterinarian at the TBRI.

### TMAdV Cultivation

The 4 human cell lines (HEK293, CaCo-2, A549, and HFDL) and 4 monkey cell lines (PMK, B95a, Vero, and BSC-1) used in this study have been previously established and are routinely maintained at the Viral and Rickettsial Disease Laboratory (VRDL) branch of the California Department of Public Health for use in viral diagnostic studies [27], [28]. Viral supernatants for cell culture passaging or the generation of infectious stocks were subjected to 3 freeze-thaw cycles and clarified by centrifugation for 10 min×4000* g*. After cells achieved 80–90% confluency, cell culture media were changed to maintenance media with 2% FBS and were inoculated with 100 µL of passaged viral supernatant. Viral replication was monitored over 14 days by visual inspection under light microscopy for cytopathic effect (CPE).

Early passages of TMAdV were not suitable for generation of infectious viral stocks for marmoset inoculation because of poor growth and insufficient titers in both monkey and human cell lines [6]. Thus, *in vivo* work with experimental marmoset infection using wild-type or early passages of TMAdV, while desirable, was not practical. Robust growth was only observed after passage 7 in A549 cells, with the appearance of the 3202 C→G in the TMAdV E1B-55k resulting in the 388P→R amino acid change (Fig. 1), reflecting TMAdV adaptation. Viral stocks at titers of ∼10^7^ TCID_50_/mL were prepared from passage 10 TMAdV initially isolated in BSC-1 and PMK cells and serially passaged in A549 cells (Fig. 1A). Infectious viral stocks were quantitated by an end-point dilution assay.

### Deep Sequencing of TMAdV

200 µL of lung swab sample in universal transport media or 100 µL of viral supernatant were passed through a 0.22 µm filter (Millipore, Temecula, CA) and total nucleic acid extraction was performed using commercially available kits according to the manufacturer’s instructions (Qiagen, Valencia, CA). Deep sequencing libraries were then prepared using a variation of the TruSeq protocol (Illumina) as described previously [6]. Raw 100-bp sequence reads were stripped of adapter and primer sequences and aligned to the reference TMAdV genome (accession number HQ913600) using BLASTn (word size = 11, E-value = 1×10^−10^) [29]. Using Geneious software [30], reads were then trimmed to exclude low quality bases or regions and mapped to the TMAdV genome. The consensus sequences were then compared to the reference TMAdV genome, with the requirement of no ambiguity at each discrepant nucleotide position.

### Experimental Infection of Marmosets with TMAdV

Three healthy adult female marmosets, 5.5 years of age and ranging in weight from 392 to 422 g, were obtained from the Southwest National Primate Research Center at the TBRI. Random screening of animals from the colony by antibody testing had been previously performed, with no viruses other than GBV-A (GB virus A) and MLCV (marmoset EBV-like lymphocryptovirus) detected. Individual screening was not performed for the 3 selected animals. In particular, screening for TMAdV was not performed because there was no *a priori* evidence that this virus naturally infected captive common marmosets. One additional female marmoset 5.5 years of age and weighing 405 g was used as a control. On day 0, three animals were transferred to a BSL-2/3 facility and then intranasally inoculated with 10^5^ TCID_50_^/^mL of virus diluted in saline, while the remaining control animal received an equivalent volume of diluted cell culture medium. At predetermined time intervals, animals were sedated and blood and nasal swab samples were collected for viral analysis. Animals began to exhibit clinical signs at days 5–10 post-inoculation and were closely observed for evidence of clinical deterioration. However, as all 3 animals recovered quickly, by day 15, two of the 3 experimentally infected marmosets were randomly selected to be euthanized. The remaining experimentally infected marmoset and control marmoset were re-inoculated with TMAdV or control media, respectively, and observed for an additional 21 days prior to euthanasia on day 36. The purpose of the re-inoculation was to assess whether repeated challenge with TMAdV would result in more severe infection or whether prior infection by TMAdV conferred any degree of protection from subsequent exposure to the virus.

### Histology

Samples of aseptically removed tissues were fixed in 10% neutral buffered formalin and embedded in paraffin for histology. Paraffin-embedded tissues were cut in 5 µm sections, deparaffinized, and stained with hematoxylin and eosin (H&E) prior to visualization by light microscopy. Additional samples were freshly frozen in liquid nitrogen and kept stored in a −80°C freezer until analyzed. Histologic sections were evaluated independently by two board-certified veterinary pathologists and the degree of tissue inflammation subjectively graded as mild, moderate, or severe.

### PCR Analysis

Nasal swabs, serum, and tissues from experimentally infected and control marmosets were screened for TMAdV using a qPCR assay from the adenoviral IVα2 gene with standard curve analysis as previously described [6]. Negative results were then screened further using a more sensitive nested PCR assay from the hexon gene. PCR amplicons were confirmed to be TMAdV by Sanger sequencing. Primers used for the PCR assays are listed in Table S3.

### Immunofluorescent Staining

Frozen tissues were cut in 4 µm sections, fixed in acetone for 20 minutes at room temperature, and stained with murine monoclonal antibodies to adenovirus 2 hexon (Diagnostic Hybrids, Athens, OH) conjugated to FITC at 37°C for 30 minutes. These antibodies have previously been shown to broadly detect a variety of mammalian adenoviruses [31]. Staining was visualized using an epifluorescence microscope.

### Nucleotide Sequence Accession Numbers

GenBank accession numbers for the adenoviral sequences in Fig. 1C are as follows: HAdV-2, AC_000007; HAdV-3, DQ086466; HAdV-4, AY458656; HAdV-5, AC_000008; HAdV-9, AJ854486; HAdV-40, NC_001454; HAdV-52, DQ923122; SAdV-3, NC_006144; SAdV-18, FJ025931; SAdV-49, NC_015225; TMAdV (titi monkey adenovirus), HQ913600. Deep sequencing reads corresponding to wild-type, passage 4, and passage 10 TMAdV have been submitted to the NCBI Sequence Read Archive (accession number pending).

## Supporting Information

Table S1 (PDF)

Table S2 (PDF)

Table S3 (PDF)
