# Supplementary material for: Experimental Cross-Species Infection of Common Marmosets by Titi Monkey Adenovirus
Source: PLoS One. 2013 Jul 24;8(7):e68558. doi: 10.1371/journal.pone.0068558 (PMC3722195; doi:10.1371/journal.pone.0068558)
Supplement: Table S1 — (PDF) [file pone.0068558.s001.pdf]

TMAdV Pneumonia	3202 C→G Mutation	Confirmation by Sequencing
serum sample from dead titi monkey with TMAdV pneumonia	absent	1 of 1 (direct sequencing)
BSC-1 supernatant, passage 1	absent	1 of 1 (direct sequencing)
A549 supernatant, passage 4	absent	3 of 3 (cloning → sequencing)
A549 supernatant, passage 7	present	6 of 6 (cloning → sequencing)
A549 supernatant, passage 10	present	9 of 9 (cloning → sequencing)
A549 supernatant, passage 13	present	12 of 12 (cloning → sequencing)
marmoset 29012, nasal swab, day 2	present	24 of 24 (cloning → sequencing)
marmoset 29012, nasal swab, day 31	present	5 of 5 (cloning → sequencing)
marmoset 29019, nasal swab, day 2	present	7 of 7 (cloning → sequencing)
marmoset 29020, nasal swab, day 2	present	7 of 7 (cloning → sequencing)

Table S1. Presence or absence of the 3202 C→G mutation in passaged TMAdV
