# Supplementary material for: Experimental Cross-Species Infection of Common Marmosets by Titi Monkey Adenovirus
Source: PLoS One. 2013 Jul 24;8(7):e68558. doi: 10.1371/journal.pone.0068558 (PMC3722195; doi:10.1371/journal.pone.0068558)
Supplement: Table S2 — (PDF) [file pone.0068558.s002.pdf]

Category	Scoring Criteria
Weight loss	0: no change from baseline 2: 5–10%↓ 3: 11–15%↓<br>4: 16 – 20%↓
Temperature / fever	0: no change from baseline 1: >2°F 2: >3°F 4: >5°F
Responsiveness	0: active 2: mild unresponsiveness, becomes active when approached 3: moderate unresponsiveness, lethargic (requires prodding), weakness 5: severe unresponsiveness, moribund
Recumbency	0: active 1: occasional prostration 2: persistent prostration but rises when approached 5: prostate
Activity:	0 = normal, active and alert 3 = abnormal, reduced activity
Dyspnea	0: normal breathing 3: labored 5: agonal
Petechiae or rash	0: none present 3: petechiae or rash present
Bleeding at venipuncture site	0: none 3: present
Bleeding other than venipuncture site	0: none 3: present
Nasal discharge	0: not present 3: visible discharge (copious)
Eyes	0: normal 1: discharge 3: partially closed 4: closed
Cubes eaten	0: ate 5-4 1: ate 4-3 2: ate 3-2 3: ate 2-1 4: ate 0-1 5: none
Stool	0: normal 2: diarrhea 4: reduced volume 5: no stool present
Fluid intake	0: drinking 2: reduced intake 3: not drinking
Dehydration (skin tent)*	0: test not done 1: 3 secs 3: 4 secs and up
TOTAL**	

* Skin tent test is performed during sedation time points.

** Total clinical score is determined by adding up all clinical scores by category. If total score is >20, animal is considered “terminally ill” and should be euthanized

Table S2. Scoring system used for assessing clinical symptoms in marmosets experimentally infected with TMAdV
