# Supplementary material for: Experimental Cross-Species Infection of Common Marmosets by Titi Monkey Adenovirus
Source: PLoS One. 2013 Jul 24;8(7):e68558. doi: 10.1371/journal.pone.0068558 (PMC3722195; doi:10.1371/journal.pone.0068558)
Supplement: Table S3 — (PDF) [file pone.0068558.s003.pdf]

Name	Sequence	Purpose
TMAdV-F	GTGACGTCATAGTTGTGGTC	real-time qPCR screening for TMAdV
TMAdV-R	CTTCGAAGGCAACTACGC	real-time qPCR screening for TMAdV
Marmoset-GAPDH-300F	CGAGATCCCTCCAAAATCAA	assessing quality of nucleic acid extractions
Marmoset-GAPDH-500R	TGACGATCTTGAGGCTGTTG	assessing quality of nucleic acid extractions
TMAdV-hexon-inner-1896F*	CGCCACCAACGTGCCCATCT	nested PCR screening for TMAdV
TMAdV-hexon-inner-2088R*	GATGGAGACGCGGCGGAAGG	nested PCR screening for TMAdV
TMAdV-hexon-outer-1882F*	CCTATCCCGGCCAACGCCAC	nested PCR screening for TMAdV
TMAdV-hexon-outer-2171R*	CCGTCCACGGCCCGTTTGAT	nested PCR screening for TMAdV
HQ913600-E1B55Kfrag-3138F	GAGTGTTTGTGCTTTTTACTGTGCCG	assessing 3202 C→G mutation
HQ913600-E1B55Kfrag-3272R	GATGTGGATGGTACGCAGGGTCT	assessing 3202 C→G mutation

Table S3. PCR primers used in this study.

*Conditions for the first-round 25 μL nested PCR reaction using TaKaRa Ex Taq™ polymerase (Takara Bio, Mountain View, CA) were 35 cycles of 98°C for 10 s, 56°C for 30 s, and 72°C for 30 s. After the first-round reaction, a 0.5 μL aliquot was used as the template for the second-round reaction at the same PCR cycling conditions.
